# Prophylactic antibiotic regimen and risk of surgical site infection in patients undergoing hysterectomy for benign disease: a retrospective cohort study

**DOI:** 10.3389/frph.2026.1838024

**Published:** 2026-05-21

**Authors:** Camila Andrea Camargo Rodríguez, Dayanna Pinto-Martínez, Víctor S. Rangel, Stephanie Flórez Herrera, Leonardo Gómez Polania

**Affiliations:** 1 Instituto de la Mujer, Hospital Universitario Mayor-Méderi, Bogotá, Colombia; 2 Universidad del Rosario, Bogotá, Colombia; 3 Clinical Investigation Group, Universidad del Rosario, Bogotá, Colombia

**Keywords:** antibiotic prophylaxis, cefazolin, hysterectomy, metronidazole, surgical wound infection

## Abstract

**Objective:**

To evaluate the association between prophylactic antibiotic regimen (cefazolin alone vs. cefazolin plus metronidazole) and the occurrence of surgical site infections (SSIs), as well as infection severity and management, in patients undergoing hysterectomy for benign disease.

**Methods:**

A retrospective cohort study was conducted at a tertiary care center, including women undergoing hysterectomy for benign indications. Patients receiving cefazolin alone were compared with those receiving cefazolin plus metronidazole. The primary outcome was overall SSIs. Secondary outcomes included infection severity (superficial, deep, organ/space) and type of management (medical vs. surgical). Crude associations were estimated using contingency tables, and adjusted odds ratios were obtained through multivariable logistic regression including clinically relevant covariates.

**Results:**

A total of 1,069 patients were included, of whom 512 received cefazolin alone and 557 received cefazolin plus metronidazole. In the adjusted analysis, the combined regimen was not associated with a reduced risk of SSIs (aORs: 1.02; 95% CI: 0.60–1.76; *p* = 0.93). Among patients with SSIs (*n* = 58), the use of cefazolin plus metronidazole was associated with lower infection severity (aORs: 0.20; 95% CI: 0.05–0.76; *p* = 0.01) and a reduced likelihood of requiring surgical management (aORs: 0.11; 95% CI 0.02–0.49; *p* = 0.007).

**Conclusion:**

The addition of metronidazole to cefazolin was not associated with a reduction in overall SSIs incidence after hysterectomy for benign disease. However, it was associated with lower infection severity and reduced need for surgical reintervention among patients who developed SSIs, suggesting a potential benefit in reducing postoperative morbidity.

## Introduction

Surgical site infections (SSIs) are defined by the Centers for Disease Control and Prevention (CDC) as any infection that occurs after surgery involving the surgical incision or the organ/space manipulated during the procedure, and that develops within 30 days following the operation or within one year when prosthetic material has been implanted (1).

The incidence of SSIs has been shown to be higher in gynecologic and obstetric procedures compared with other surgical specialties (2). Despite advances in preventive strategies, SSIs remain one of the most frequent postoperative complications, representing a significant source of morbidity, as well as reconsultations, hospital readmissions, and increased healthcare costs (3). Hysterectomy is among the gynecologic procedures most affected by SSIs, with reported rates ranging from 2% to 2.7%, likely related to its high frequency and the inherent risk of postoperative infectious complications (4). This is particularly relevant considering that more than 600,000 hysterectomies are performed annually in countries such as the United States (5).

The administration of prophylactic antibiotics prior to surgery represents one of the most important strategies for the prevention of SSIs, as it has been shown to reduce their incidence by approximately 47% to 56%, as well as associated complications (6). As antimicrobial therapies have evolved, prophylactic antibiotic regimens have been progressively refined and adapted to the epidemiological patterns of SSIs, taking into account the type of surgical procedure and the baseline risk profile associated with each technique (7). Among the most commonly used prophylactic antibiotic regimens is the administration of a preoperative dose of cefazolin 2 g. This choice is supported by robust evidence demonstrating its effectiveness, appropriate duration of action, antimicrobial spectrum against the most common surgical pathogens, as well as its favorable safety profile and low cost (8).

Literature reviews have reported reductions in SSIs incidence when comparing cefazolin alone with cefazolin plus metronidazole, with decreases that may reach, for example, from 7.9% to 3.3% when intravenous metronidazole is used (9). Therefore, the aim of the present study was to evaluate the association between prophylactic antibiotic regimen (cefazolin alone vs. cefazolin plus metronidazole) and the occurrence of SSIs in patients undergoing hysterectomy for benign disease in a tertiary care center.

## Materials and methods

### Study design

We conducted an observational, retrospective cohort study including patients who underwent hysterectomy for benign disease between 2021 and 2025 at Hospital Universitario Mayor Méderi, Bogotá, Colombia. Clinical and perioperative data were extracted from electronic medical records, with follow-up extending up to 30 days after surgery to assess the occurrence of SSIs. Ethical approval was obtained from the Human Research Ethics Committee of Hospital Universitario Mayor Méderi (Approval No. CEISH-2025023).

### Eligibility criteria and data collection

Women aged ≥18 years who underwent hysterectomy were eligible for inclusion. Only patients undergoing hysterectomy for benign gynecologic conditions (specifically uterine fibroids, abnormal uterine bleeding, or adenomyosis) were included, with subsequent confirmation of benign pathology on histopathological examination. Patients were classified according to the prophylactic antibiotic regimen received into two groups: those who received cefazolin 2 g administered intravenously (non-exposed group) and those who received cefazolin 2 g plus metronidazole 500 mg administered intravenously (exposed group). Patients who developed *de novo* hypersensitivity reactions consistent with type I allergic responses during antibiotic administration, without a prior history of allergy, were excluded. Additionally, patients requiring postoperative antibiotic therapy due to clinical indications, as well as those with any documented preoperative infection regardless of etiology, were excluded from the analysis. SSIs was defined according to the criteria established by the CDC and classified as superficial, deep, or organ/space infection (1). Data collection and management were performed using REDCap.

### Statistical analyses

Sample size estimation was conducted to support both the primary association analysis and the development of a multivariable logistic regression model. The primary outcome was SSIs, with an expected baseline incidence of approximately 5%. Assuming a two-sided alpha of 0.05% and 80% power, a minimum of 199 patients per group (total *n* = 398) was required to detect a clinically relevant difference between exposure groups. To ensure adequate model stability and reduce the risk of overfitting in multivariable analyses, a larger sample size of approximately 1,000 patients was targeted, allowing for an appropriate events-per-variable ratio.

Continuous variables were summarized as mean ± standard deviation or median with interquartile range (IQR), according to their distribution, and categorical variables as absolute frequencies and percentages. Normality was assessed using the Shapiro–Wilk test in conjunction with graphical inspection. Comparisons between groups were performed using the independent-samples Student's *t* test for normally distributed variables or the Wilcoxon rank-sum test otherwise. Categorical variables were compared using Fisher's exact test. The proportion of missing data for the variables included in the analysis was low (<6% for all covariates). Given this minimal level of missingness, no formal statistical methods for handling missing data were applied.

The association between prophylactic antibiotic regimen and the occurrence of SSIs was evaluated using multivariable logistic regression. Adjusted odds ratios (aORs) and 95% confidence intervals (95% CI) were estimated, including clinically relevant covariates selected *a priori*: age, body mass index, diabetes mellitus, endometriosis, uterine volume, intraoperative blood loss, and operative time.

Among patients who developed SSIs, infection severity was analyzed using an ordinal logistic regression model under the proportional odds assumption, which was evaluated prior to model interpretation. The type of management (medical vs. surgical) was assessed using binary logistic regression. All analyses were performed using Stata version 17.

## Results

### Participants

**Figure 1:**
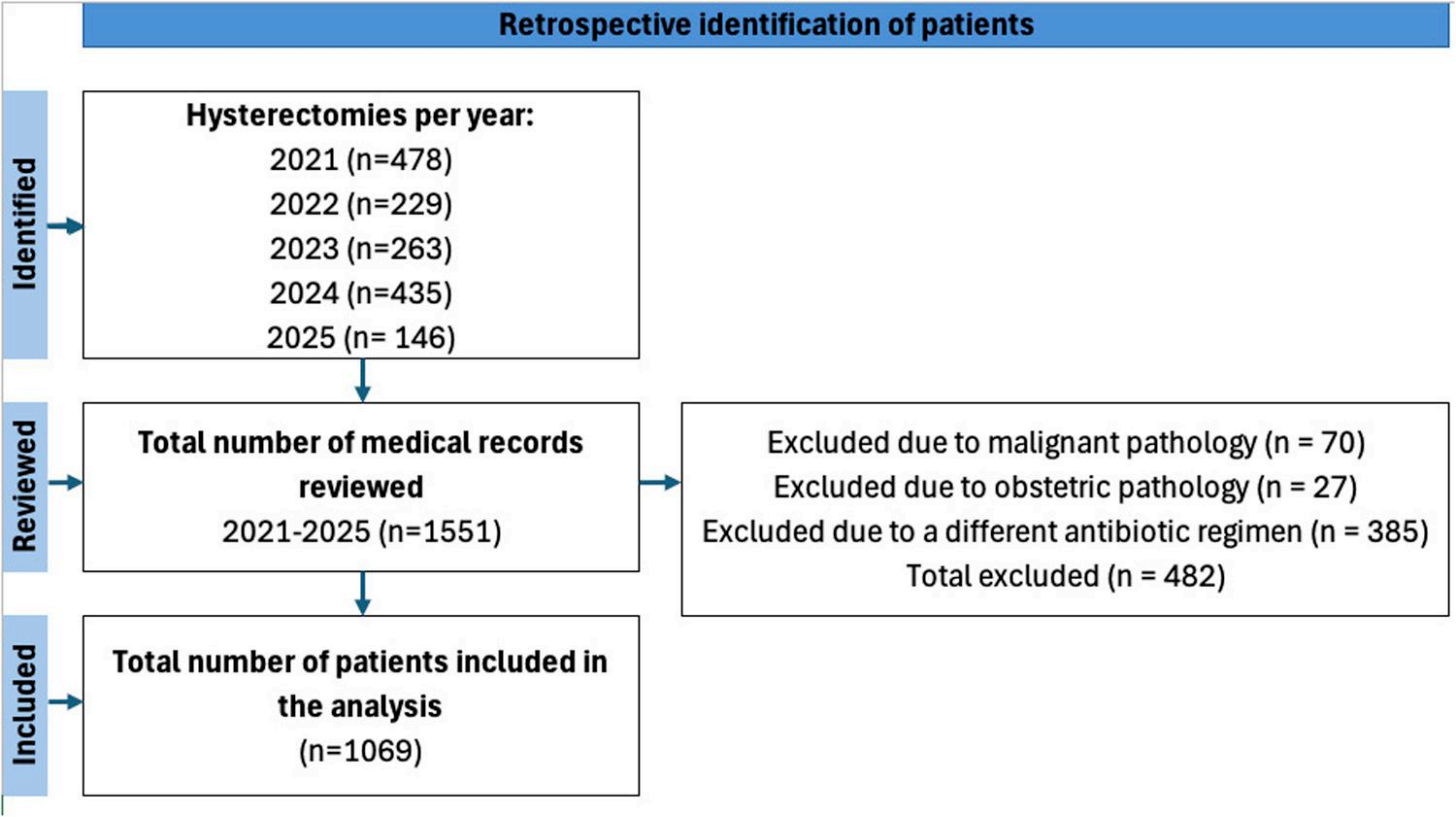
Flowchart summarizing the patient selection process for the retrospective cohort of women undergoing hysterectomy.

A total of 1,069 patients were included, of whom 512 (47.9%) received antibiotic prophylaxis with cefazolin 2 g, while 557 (52.1%) received the combined regimen of cefazolin 2 g plus metronidazole. Figure 1 presents the flow diagram corresponding to the sample selection process. Age was comparable between both groups, with a median of 46 years in the cefazolin group (IQR: 42–51) and 46 years in the combined group (IQR: 41–50), with no statistically significant differences (*p* = 0.40). Similarly, body mass index did not differ between antibiotic regimens, with a median of 26.25 kg/m² in the cefazolin group (IQR: 23.6–29.5) and 26.25 kg/m² in the combined group (IQR: 23.7–29.5; *p* = 0.80).

Regarding comorbidities, diabetes mellitus was significantly more frequent in the group that received cefazolin plus metronidazole compared with the group treated with cefazolin alone (7.9% vs. 4.5%, respectively; *p* = 0.02). No relevant differences were observed between groups in the prevalence of hypertension, anticoagulation use, or endometriosis. In contrast, differences were identified in the presence of adenomyosis (42% vs. 13%, *p* < 0.001), abnormal uterine bleeding (86% vs. 77%, *p* < 0.001), and uterine leiomyomatosis (89% vs. 83%, *p* = 0.004), all of which were more frequent in the group that received the combined antibiotic regimen.

Uterine volume tended to be higher in the group treated with cefazolin plus metronidazole, with a median of 270.5 cm³ (IQR: 142.0–504.5), compared with the group that received cefazolin alone, which had a median of 250.5 cm³ (IQR: 125.0–469.0); however, this difference did not reach statistical significance (*p* = 0.06). Perioperative variables, including preoperative hemoglobin, intraoperative blood loss, operative time, and length of hospital stay, were similar between both groups, with no significant differences. Finally, the ASA classification showed significant differences between groups (*p* = 0.002), with a higher proportion of patients classified as ASA ≥3 in the group treated with cefazolin alone, suggesting a slightly more complex baseline clinical profile in this group. These results are fully detailed in Table 1.

**Table 1:** Baseline sociodemographic, clinical, and surgical characteristics of patients according to the antibiotic prophylaxis regimen used.

Characteristics	Cefazolin 2 g	Cefazolin 2 g + Metronidazole	*p*-value
	(*n* = 512)	(*n* = 557)	
Age, median (IQR)	46.00 (42.00, 51.00)	46.00 (41.00, 50.00)	0.40
BMI, kg/m², median (IQR)	26.25 (23.60, 29.50)	26.25 (23.70, 29.50)	0.80
Parity, *N* (%)			0.20
Nulliparous	79 (15%)	67 (12%)	
Multiparous	431 (84%)	487 (87%)	
Diabetes mellitus, *N* (%)	23 (4.5%)	44 (7.9%)	**0** **.** **02**
Chronic hypertension, *N* (%)	96 (19%)	110 (20%)	0.90
Anticoagulation, *N* (%)	16 (3.1%)	12 (2.2%)	0.30
Endometriosis, *N* (%)	36 (7%)	32 (5.7%)	0.50
Adenomyosis, *N* (%)	64 (13%)	234 (42%)	**<0** **.** **001**
Abnormal uterine bleeding, *N* (%)	394 (77%)	481 (86%)	**<0** **.** **001**
Uterine leiomyomatosis, *N* (%)	427 (83%)	498 (89%)	**0** **.** **004**
Previous surgeries, mean (SD)	1.24 ± 1.13	1.49 ± 1.37	**0** **.** **007**
Uterine volume (cc), median (IQR)	250 (125, 469)	270.5 (142, 504.5)	0.06
Baseline hemoglobin (g/dL), median (IQR)	13 (10.8, 14.4)	13 (10.1, 14.3)	0.50
Blood loss (mL), median (IQR)	200 (110, 300)	200 (200, 300)	0.50
Operative time (min), median (IQR)	94 (78, 118)	93 (75, 115)	0.40
Length of hospital stay (days), median (IQR)	2 (1,2)	2 (1,3)	0.30
ASA classification, *N* (%)			**0** **.** **002**
1 or 2	443 (87%)	484 (87%)	
≥3	63 (12%)	49 (8.8%)	
Surgical approach, *N* (%)			**<0** **.** **001**
Abdominal	357 (70%)	466 (84%)	
Laparoscopic	92 (18%)	61 (11%)	
Vaginal	63 (12%)	30 (5.4%)	
Intraoperative complications, *N* (%)	33 (6.4%)	52 (9.3%)	0.09

Statistical significance indicated in bold.

ASA, American society of anesthesiologists physical status classification; BMI: body mass index; IQR: interquartile range; SD: standard deviation.

### Outcomes

The overall incidence of SSIs was low and similar between both antibiotic regimens, as described in Table 2. No significant difference was observed in the occurrence of SSIs between the combined regimen (cefazolin plus metronidazole) and cefazolin alone (aORs = 1.02; 95% CI: 0.60–1.76; *p* = 0.93) (Table 3). Among the covariates included in the model, endometriosis was identified as an independent risk factor for the development of SSIs (aORs = 2.60; 95% CI: 1.07–5.63; *p* = 0.02), whereas diabetes mellitus showed a trend toward increased risk, although it did not reach statistical significance (aORs = 2.00; 95% CI: 0.76–4.67; *p* = 0.13). Additional analyses, including confounding assessment and a sensitivity analysis using a propensity score approach, yielded consistent findings, supporting the robustness of the results.

**Table 2:** Association between the antibiotic prophylaxis regimen and surgical site infection according to infection type and management.

Variables	Cefazolin 2 g	Cefazolin 2 g + Metronidazole	*p*-value
	(*n* = 512)	(*n* = 557)	
Surgical site infection, *N* (%)			0.90
No	485 (95%)	526 (94%)	
Yes	27 (5.3%)	31 (5.6%)	
Type of surgical site infection, *N* (%)			**0** **.** **02**
Superficial infection	12 (44%)	23 (74%)	
Deep infection	2 (7.4%)	0 (0%)	
Organ/space infection	13 (48%)	8 (26%)	
Management of surgical site infection, *N* (%)			**0** **.** **004**
Medical management	14 (52%)	27 (87%)	
Surgical management	13 (48%)	4 (13%)	

Statistical significance indicated in bold.

Data are presented as absolute frequencies and percentages. Comparisons between groups were performed using Fisher's exact test. The analysis of Surgical site infections (SSIs) type and management was restricted exclusively to patients who developed SSIs.

**Table 3:** Association between the antibiotic prophylaxis regimen and surgical site infection according to overall incidence and requirement for surgical management.

Variables	SSIs (*n* = 1,069)		
	cOR (95% CI)	aOR (95% CI)	*p*-value
Cefazolin		**Ref.**	
Cefazolin + metronidazole	1.05 (0.62–1.79)	1.02 (0.60–1.76)	0.93
Age		1.00 (0.97–1.04)	0.77
Diabetes mellitus		2.00 (0.76–4.67)	0.13
BMI, kg/m²		1.03 (0.97–1.09)	0.27
Endometriosis		**2.60** **(1.07–5.63)**	**0** **.** **02**
Uterine volume		1.00 (1.00–1.00)	0.82
Blood loss (mL)		1.00 (1.00–1.00)	0.44
Operative time (min)		1.00 (1.00–1.01)	0.17
Variables	**Surgical management of SSIs (n = 58)**		
	**cOR (95% CI)**	**aOR (95% CI)**	***p*-value**
Cefazolin		**Ref.**	
Cefazolin + metronidazole	**0.15 (0.04–0.58)**	**0.11** **(0.02–0.49)**	**0** **.** **007**

Statistical significance indicated in bold.

Data are presented as crude odds ratios (cOR) and adjusted odds ratios (aOR) with their corresponding 95% confidence intervals (95% CI). The aORs were estimated using multivariable logistic regression models. The model evaluating overall SSIs was adjusted for age, diabetes mellitus, body mass index, endometriosis, uterine volume, intraoperative blood loss, and operative time. The model assessing infection management was estimated in the subgroup of patients who developed SSI and was adjusted for the same covariates. BMI: body mass index; SSIs: surgical site infections.

### Severity of SSIs by antibiotic regimen

When analyzing exclusively the patients who developed SSIs, clinically relevant differences were observed in the severity of the infectious event according to the antibiotic regimen used. The distribution by type of infection showed that superficial infections were similar between groups. In contrast, deep infections occurred only in the group that received cefazolin alone, with no cases in the group treated with cefazolin plus metronidazole. Consistently, organ/space infections were less frequent in the group that received the combined regimen (see Table 2). The use of cefazolin plus metronidazole was associated with a reduction in the odds of developing more severe surgical site infections compared with cefazolin alone (aORs = 0.20; 95% CI: 0.05–0.76; *p* = 0.01), indicating a lower likelihood of progression to deep or organ/space infections.

### Surgical management of SSIs by prophylactic regimen

Analysis of infection management revealed differences in the need for surgical reintervention according to the prophylactic antibiotic regimen. Among patients who developed SSIs, those who received combined prophylaxis with cefazolin plus metronidazole had a lower likelihood of requiring surgical management compared with patients treated with cefazolin alone. In the multivariable logistic regression model, the use of cefazolin plus metronidazole was independently associated with a reduced probability of requiring surgical intervention (aORs = 0.11; 95% CI: 0.02–0.49; *p* = 0.007).

## Discussion

### Principal findings

Overall, these findings suggest that, although the addition of metronidazole does not significantly modify the overall incidence of SSIs, it may be associated with reduced clinical severity of infections. This association may be reflected in a lower likelihood of progression to more severe forms of infection and a reduced need for surgical management, which could translate into a lower postoperative morbidity burden.

### Results in the context of existing knowledge

Among the largest studies evaluating outcomes similar to those of the present work, the study by Till et al. stands out. They conducted a multicenter retrospective cohort study using data from the Michigan Surgical Quality Collaborative to assess the effectiveness of the combination of cefazolin plus metronidazole in SSIs in patients undergoing hysterectomy, compared with cefazolin alone or second-generation cephalosporins. The study included 18,255 women undergoing hysterectomy for both benign and malignant indications, with 30-day follow-up. The overall SSIs incidence was 1.8%, being lower in the group treated with cefazolin plus metronidazole (1.4%) compared with cefazolin (1.8%) and second-generation cephalosporins (2.1%). In the multivariable analysis, regimens without metronidazole were associated with a higher risk of infection compared with the combined therapy (cefazolin: OR: 2.30; 95% CI: 1.06–4.99; *p* = 0.03; second-generation cephalosporins: OR: 2.31; 95% CI: 1.21–4.41; *p* = 0.01). These findings were confirmed by propensity score–matched analyses, in which the SSIs incidence was significantly lower in the combined group (0.8% vs. 1.5%; *p* = 0.008) (10).

Comparable investigations have been reported in the literature, although they have been conducted predominantly in oncologic populations. Gorman et al. performed a multicenter retrospective cohort study to determine whether the addition of metronidazole to standard antibiotic prophylaxis (cefazolin or second-generation cephalosporins) reduces the risk of SSIs in patients undergoing hysterectomy for gynecologic cancer. The study included 1,055 patients treated between 2020 and 2022 across four centers, with an overall SSIs incidence of 3.2% (*n* = 34). In the bivariate analysis, the infection rate was lower in the group that received metronidazole (2.1% vs. 4.3%; *p* = 0.04), suggesting a potential benefit. However, in the multivariable analysis using mixed-effects logistic regression, adjusted for clinically relevant variables such as diabetes, smoking, and surgical approach, the addition of metronidazole was not significantly associated with a reduction in SSIs risk (OR: 0.5; 95% CI, 0.3–1.1; *p* = 0.09) (11).

Knisely et al. also conducted a retrospective cohort study in a tertiary oncologic center including 3,343 patients, comparing similar antibiotic prophylactic regimens. In the hysterectomy subgroup, the SSIs rate decreased significantly from 4.9% to 2.8% (*p* = 0.03). In multivariable analysis, the intervention was associated with a significant reduction in SSIs risk (aORs: 0.49; 95% CI, 0.38–0.63; *p* < 0.001). In sensitivity analyses, the use of cefazolin plus metronidazole was associated with a lower incidence of SSIs compared with cefazolin alone (2.3% vs. 4.5%; *p* = 0.01), as well as a reduced risk of infection (aORs: 0.40; 95% CI, 0.30–0.53; *p* < 0.001) (12). These findings suggest that the impact of combined antibiotic prophylaxis on SSIs outcomes may vary according to the underlying patient population and baseline risk factors associated with different types of hysterectomy.

### Clinical implications

**Figure 2:**
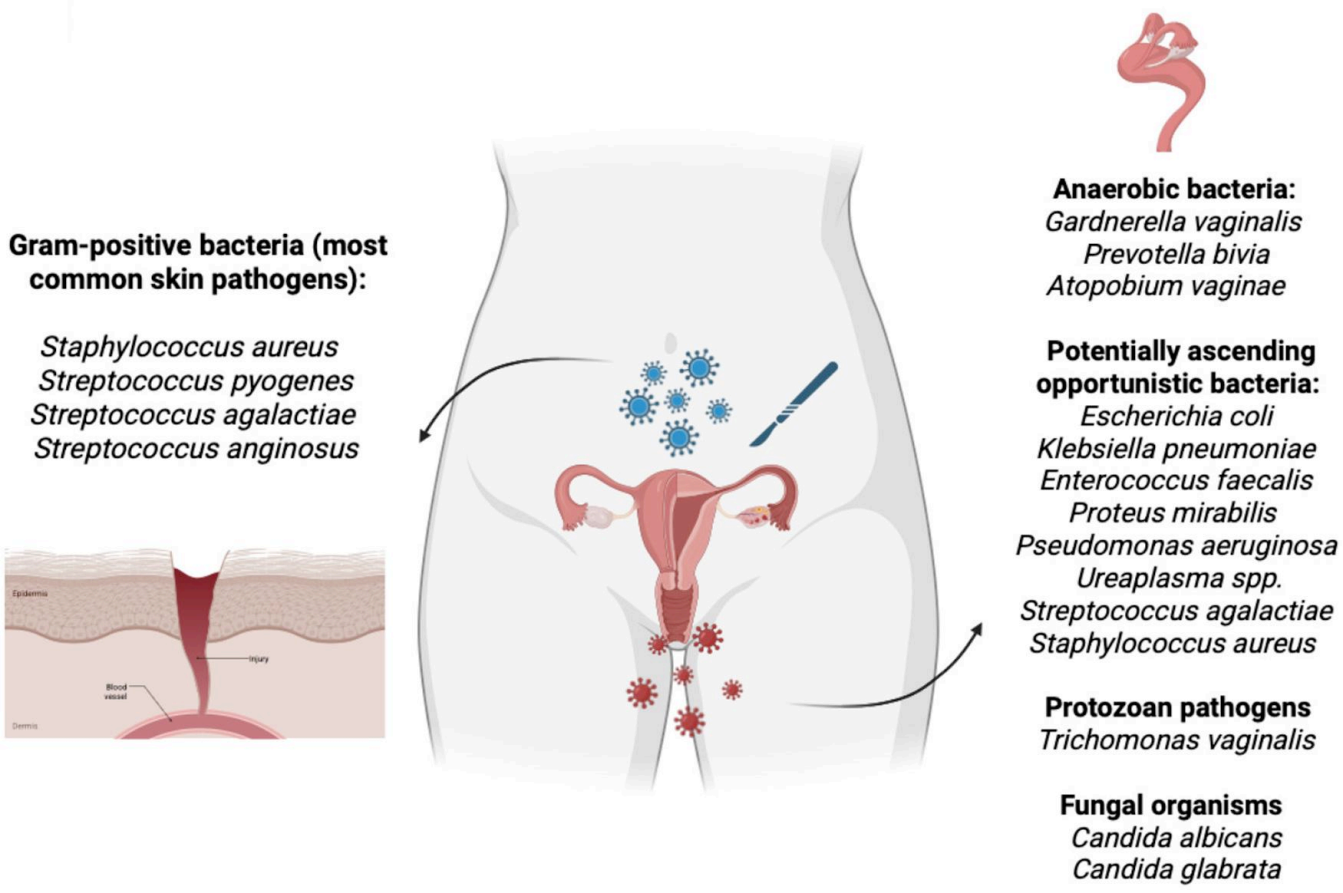
Skin and vaginal microbiota as potential sources of surgical site infection in hysterectomy. The left panel illustrates the most common pathogenic microorganisms of the skin, which may contaminate the surgical field during incision or tissue manipulation. These organisms are predominantly gram-positive bacteria. The right panel depicts microorganisms with potential pathogenicity within the vaginal environment, including anaerobic bacteria, opportunistic ascending bacteria, protozoa, and fungal organisms. These microorganisms may ascend from the vaginal microbiota and contribute to postoperative infectious complications in gynecologic procedures.

This finding suggests that anaerobic coverage with metronidazole may not prevent the initial infection but may limit its progression to deeper or more complex forms. From a pathophysiological perspective, this is plausible, as most microorganisms isolated from vaginal cuff infections include a high proportion of anaerobes (10, 13). Therefore, the addition of metronidazole may influence the microbiological profile (see Figure 2) and clinical severity of the infectious event (14, 15). Additionally, metronidazole plays a key role in the treatment of bacterial vaginosis and in controlling the overgrowth of anaerobic vaginal microbiota, a condition that may be present in up to one in three women (13). SSIs following hysterectomy in patients with bacterial vaginosis may reach an incidence of up to 34%. Randomized clinical trials have demonstrated that preoperative administration of metronidazole significantly reduces the rate of vaginal cuff infection in this group of patients (9).

Several international clinical practice guidelines continue to recommend cefazolin monotherapy as the standard antibiotic prophylaxis regimen in gynecologic surgery (16). However, the findings of the present study, together with the previously cited evidence, suggest the need to reevaluate the role of combined regimens in the prevention of SSIs, as well as to promote a deeper understanding of local and evolving antibiotic resistance patterns to better inform prophylactic strategies.

## Strengths and limitations

This study has several strengths that enhance the validity and clinical relevance of its findings. First, it included a relatively large sample size of more than 1,000 patients undergoing hysterectomy for benign disease in a tertiary care center, allowing for adequate statistical power and a sufficient number of events to perform multivariable analyses. Second, the study was conducted in a real-world clinical setting, which increases the external validity and applicability of the results to routine clinical practice. Another important strength is the comprehensive evaluation of outcomes beyond overall SSIs incidence. The study assessed not only the occurrence of infection but also clinically meaningful outcomes such as infection severity and need for surgical management.

Despite these strengths, several limitations should be considered when interpreting the results. First, the retrospective observational design inherently limits causal inference and may be subject to residual confounding, despite adjustment for multiple covariates. Unmeasured factors, such as surgeon experience, intraoperative contamination, or adherence to perioperative protocols, may have influenced the observed outcomes. Second, the relatively low incidence of SSIs, although consistent with previous reports, may have limited the statistical power for certain analyses, particularly subgroup evaluations such as infection severity and management strategies, which were restricted to patients who developed SSIs. Third, the study was conducted at a single tertiary care center, which may limit the generalizability of the findings to other settings with different patient populations, surgical practices, or antimicrobial resistance patterns.

## Conclusions

In this retrospective cohort study, the addition of metronidazole to cefazolin for surgical prophylaxis in patients undergoing hysterectomy for benign disease was not associated with a reduction in the overall incidence of SSIs. However, the combined regimen was associated with lower infection severity and a reduced need for surgical management among patients who developed infection. These findings suggest that while extended antibiotic coverage may not impact the occurrence of infection, it could influence its clinical course and postoperative morbidity. Further prospective studies are warranted to confirm these results and to better define the role of combined antibiotic prophylaxis in this setting.

## Data Availability

The datasets generated and/or analyzed during the current study are available from the corresponding author on reasonable request.
